# A Data Warehouse-Based System for Service Customization Recommendations in Product-Service Systems

**DOI:** 10.3390/s22062118

**Published:** 2022-03-09

**Authors:** Laila Esheiba, Iman M. A. Helal, Amal Elgammal, Mohamed E. El-Sharkawi

**Affiliations:** 1 Faculty of Computers and Artificial Intelligence, Cairo University, Giza 12613, Egypt; i.helal@fci-cu.edu.eg (I.M.A.H.); amal.elgammal@eui.edu.eg (A.E.); mohamed.elsharkawi@eui.edu.eg (M.E.E.-S.); 2 Faculty of Computing and Information Sciences, Egypt University of Informatics, New Administrative Capital, Cairo 11865, Egypt

**Keywords:** data analytics, data warehousing, decision support systems, product-service systems (PSSs), product-service systems customization, product usage data, recommender systems (RSs), sensors

## Abstract

Nowadays, manufacturers are shifting from a traditional product-centric business paradigm to a service-centric one by offering products that are accompanied by services, which is known as Product-Service Systems (PSSs). PSS customization entails configuring products with varying degrees of differentiation to meet the needs of various customers. This is combined with service customization, in which configured products are expanded by customers to include smart IoT devices (e.g., sensors) to improve product usage and facilitate the transition to smart connected products. The concept of PSS customization is gaining significant interest; however, there are still numerous challenges that must be addressed when designing and offering customized PSSs, such as choosing the optimum types of sensors to install on products and their adequate locations during the service customization process. In this paper, we propose a data warehouse-based recommender system that collects and analyzes large volumes of product usage data from similar products to the product that the customer needs to customize by adding IoT smart devices. The analysis of these data helps in identifying the most critical parts with the highest number of incidents and the causes of those incidents. As a result, sensor types are determined and recommended to the customer based on the causes of these incidents. The utility and applicability of the proposed RS have been demonstrated through its application in a case study that considers the rotary spindle units of a CNC milling machine.

## 1. Introduction

Manufacturers are attempting to fulfill orders on-demand by conducting business processes over short-term networks while considering *customer requirements* (e.g., functional, structural, environmental, and performance aspects of product design offerings), *quality of manufactured products*, *sustainability* (e.g., producing manufactured products by using economically sound processes that minimize wastes and reduce negative environmental impacts while saving energy and natural resources, designing products to achieve targeted objectives (e.g., cost, quality, reliability, etc.) using systematic approaches such as the Design for Excellence approach (aka Design for X) [1]), *time* (e.g., reducing lead times), *price*, and other dimensions [2]. Furthermore, manufacturers are competing to offer not only products but also products accompanied with services, which are referred to as “Product-Service Systems” (PSSs) [3]. PSSs provide a combined product and service offering that adds value while the product is in use. Moreover, they make certain that the customer’s usage experience does not only end with the purchase but extends much further [4].

PSS mass customization is defined as the production of products and services with near mass production efficiency to meet the needs of individual customers. PSS customization entails configuring products with varying degrees of differentiation to meet the needs of various customers. This is combined with service customization, in which customers expand configured products to include smart sensors or IoT communication devices in general to improve product usage and facilitate the transition to smart connected products. These sensors (e.g., temperature sensor, humidity sensor, vibration sensor, etc.) are embedded in products to regularly monitor physical parameters in machinery such as vibration, temperature, pressure, etc., to detect changes that may indicate a developing fault. Despite the significant gained value from adding sensors to products, the selection of the appropriate types of sensors and their adequate locations is a challenge that customers are unable to manage easily and effectively [5]. Moreover, placing sensors randomly may result in cost increases, minimizing energy savings (e.g., high power consumption), minimizing the Return on Assets (RoA) (i.e., the profit returned for each dollar held on assets) because of the selection of inefficient sensors, and less satisfied customers.

During the use phase of PSSs, a massive amount of Product Usage Information (PUI) is collected, such as product usage incidents (e.g., cracks, leaks, etc.), product service data, product operational environment, product user information, etc. [6,7,8]. We envision that PUI, particularly product usage incidents, could play a pivotal role in assisting customers (e.g., aerospace engine manufacturers) in selecting the appropriate types of sensors to install on their machines (e.g., milling machine) during the service customization process to regularly monitor machine functions and physical operating parameters. Product usage incidents data are useless and invaluable unless useful information and insights are generated from it. Analyzing product usage incidents data from similar products to the product that the customer needs to expand by adding smart sensors helps in identifying the most critical parts with the highest number of incidents and the causes of those incidents. Based on this analysis and the failure modes at hand, sensor types for monitoring these critical parts can be determined and recommended to the customer.

However, the analysis of these data is not supported by PSSs to improve data-driven decision making. Consequently, this creates a demand for the adoption of novel techniques/approaches for analyzing such data to assist customers in making informed decisions. Accordingly, this article addresses the following research questions:

- Q1: How can data analytics techniques be used to assist customers in making informed decisions during the customization of services process?
- Q2: How can PUI, particularly product usage incidents, be exploited to assist customers in making informed decisions during the customization of services process?

To answer these research questions, data analytics techniques can be employed to analyze these massive amounts of product usage incidents data collected during the PSSs’ usage phase. Data-driven decision making can be improved by analyzing these data, leading to the identification of new opportunities, more satisfied customers, and more efficient operations. Data analytics techniques are classified into three categories: descriptive, predictive, and prescriptive [9]. Prescriptive analytics is the most sophisticated type of data analytics, and it employs statistics and data mining techniques to recommend the best course of action for a given situation.

Recommender Systems (RSs), which fall under the category of prescriptive analytics, are defined as software tools that provide useful suggestions to customers while taking their requirements/preferences into account [10]. Recommender systems have gained popularity due to their applicability in several domains (e.g., e-commerce, tourism, health, e-learning, etc.) and in applications that provide personalized services [10]. There are various types of recommender systems. These types include collaborative filtering RSs [10], content-based RSs [11], knowledge-based RSs [12], hybrid RSs [11], knowledge graph-based RSs [13], and cognitive-based RSs [14].

Data warehousing (DW) is capable of providing extreme performance in managing and analyzing big data by extracting useful insights from massive amounts of data [15]. DW is defined as a repository that integrates heterogeneous data from multiple data sources into a single multi-dimensional source for the purpose of analyzing and extracting new knowledge from these data. Besides the previously mentioned RS techniques, DW techniques have been used for generating recommendations and creating RSs in many applications, such as movies, books, and tourism [15].

We anticipate that data warehousing concepts and capabilities could play an important role in assisting customers in making informed decisions during the customization of services process. Accordingly, in this paper, we propose a data warehouse-based RS that assists customers (e.g., aerospace engine manufacturer, electronic appliances manufacturer, etc.) in determining the appropriate types of sensors (e.g., temperature sensor) to install on their machines (e.g., milling machines, laser cutting machines, etc.). The proposed RS utilizes data warehousing concepts to collect and analyze large volumes of product usage information (PUI), particularly product usage incidents (e.g., cracks, leaks, etc.) from similar products to the product that the customer wishes to expand by adding smart sensors. By analyzing these data, the most critical parts with the highest number of incidents, as well as the causes of those incidents, are identified. Accordingly, based on the causes of these incidents, appropriate sensor types are determined and recommended to the customer. A case study that considers the rotary spindle units of a CNC milling machine is used to demonstrate the applicability and utility of the proposed recommendation approach and its implemented solutions.

The main contributions of this paper are:

- A snowflake schema-based dimensional model for capturing products’ usage incidents is proposed.
- The design of a data warehouse-based recommender for service customization recommendations in PSSs is proposed.
- To support the service customization recommendation process, an extension of the manufacturing blueprints models presented in [16,17] is provided.
- To ensure the applicability of the proposed approach, a web-based prototype system implementing all of the proposed RS modules has been developed.
- The performance of the proposed system is evaluated experimentally in terms of response time.

The remainder of this paper is organized as follows: Related work efforts are presented in Section 2. This is followed by presenting the case study in Section 3. Section 4 presents the architecture of the proposed data warehouse-based recommender system and its data warehouse schema components. Manufacturing blueprints are discussed in Section 5, with extensions to support the service customization recommendation process. The implementation and evaluation details are included in Section 6. Finally, Section 7 highlights the conclusion and future work.

## 2. Related Work

Related works are categorized into three directions: (i) recommendation approaches in manufacturing, (ii) domains of exploiting product usage data, and (iii) the role of data warehousing in generating recommendations.

### 2.1. Manufacturing Recommendation Approaches

Recommendation technology is a rapidly expanding research domain and is considered a hot topic in the information technology industry. Recommender Systems (RSs) are software tools that use information about the items, the users, and the interactions between users and items to suggest the most appropriate items to the users [10]. RSs have been applied in a variety of domains such as e-commerce [18,19,20], telecommunications [21], tourism [22,23], and financial services [24,25]. There are various types of recommendation techniques; the commonly used techniques are:

- Content-Based (CB) technique: Recommendations are generated based on the product’s features and user preferences. It suggests products that have similar features to the ones enjoyed by the customer in the past [11]. In content-based recommender systems, two basic techniques are used for generating and calculating recommendations. The first one uses traditional information retrieval techniques such as cosine similarity for generating recommendations. The other technique utilizes machine learning and statistical learning techniques for predicting the users’ interests from training data.
- Collaborative Filtering (CF) technique: Predicts the users’ interests based on the taste of other users [10]. This technique is divided into item-based and user-based CF approaches. However, content-based and collaborative filtering techniques have some limitations, such as the data sparsity problem, the cold start problem, the grey sheep problem, overspecialization, and limited content analysis [12]. Therefore, the knowledge-based technique emerged to address the limitations of the content-based and collaborative filtering approaches.
- Knowledge-based technique: Generates recommendations based on the domain knowledge and explicit customer requirements. It does not take into account the behavior of other users [12]. Knowledge-based techniques are classified into two techniques: the Case-Based Reasoning (CBR) technique [26] and the constraint-based technique [27]. The CBR technique uses similarity metrics for generating recommendations. The constraint-based technique, on the other hand, makes use of a recommender knowledge base that includes explicit constraints on how to relate customer requirements to product attributes. Knowledge-based techniques aid in the resolution of problems associated with CB and CF techniques, such as cold start, data sparsity, and the grey sheep problem.
- Hybrid technique: combines two or more recommendation techniques into one hybrid technique to enhance the performance of traditional techniques [10].

In the manufacturing domain, recommender systems have been utilized successfully. In [28], the authors proposed a hybrid machine learning approach for generating additive manufacturing design feature recommendations for target components during the design phase in the Additive Manufacturing (AM) domain. The proposed method integrates two algorithms: clustering and Support Vector Machine (SVM) for generating recommendations. The proposed approach was validated by applying it to a case study of designing R/C racing car components.

Influential related work efforts have utilized RSs to assist customers in identifying the manufacturing services (e.g., resources, capabilities) needed to accomplish the required manufacturing task [29,30,31,32,33,34].

Some authors have used a hybrid recommendation method that integrates social network and collaborative filtering techniques to recommend manufacturing services [29,30]. By adopting collaborative filtering and social network techniques, the authors in [29] predicted the missing Quality of Services (QoS) values of manufacturing services. Finally, the top-k manufacturing services with the highest QoS values are recommended to service consumers. While in [30], the authors utilized a Stochastic Approach for Link Structure Analysis (SALSA) to base their hybrid approach for recommending manufacturing services. SALSA was employed to select the top trustworthy enterprises. The selected top trustworthy enterprises and three similar enterprises are regarded as the influential components for calculating predicted ratings of candidate services. Eventually, personalized manufacturing services are recommended by adopting an extended user-based CF method.

In [31], the authors proposed a novel approach for predicting personalized QoS and reliable cloud manufacturing service recommendations by combining a clustering-based algorithm and a trust-aware CF approach.

The authors of [32] proposed a recommendation approach that employs a Time-aware Targeted Reconstructing Service Descriptions (T-TRSD) model for manufacturing service recommendations. The T-TRSD model is used to reconstruct descriptions of a single manufacturing service for specific requirements while taking the changing characteristics and descriptions of service composition of cloud manufacturing services into account. Eventually, manufacturing service recommendations are generated by extracting useful information from the reconstructed manufacturing service descriptions.

A deep neural network model was proposed in [33] for cloud manufacturing service recommendations. The optimal manufacturing services are recommended based on automatic learning from the customers’ previous history and their new choices.

In [34], a machine learning-based regression approach was proposed for recommending manufacturing services in the cloud manufacturing domain. The proposed approach employs a three-layer feed-forward neural network to segment customers based on historical data from previous manufacturing solution selections. The system then generates a list of ranked manufacturing solutions that meet the requirements of each customer profile.

Another stream of research work efforts has adopted advanced data analytics techniques (e.g., Deep Neural Networks (DNN)) for production quality prediction [35,36,37]. In [35], the authors proposed a prediction model that integrates a Deep Belief Neural Network with a regression model to predict product quality. A deep learning-based approach was proposed in [36] for predicting the future values of machines’ key performance indicators (e.g., Machine Mechanical Efficiency (MME)). In [37], the authors proposed a deep learning-based approach that integrates a minimal-Redundancy-Maximal-Relevance (mRMR) algorithm with a Convolutional Neural Network (CNN) to predict the quality of batch processes.

Some authors have combined data analytics techniques with Cyber-Physical Systems (CPS) technology and IoT technology to enable production visibility and traceability in Cyber-Physical Production Systems (CPPS) [38,39,40,41]. In [38], the authors utilized data analytics techniques (e.g., Complex Event Processing (CEP)) to realize production progress visibility. A methodological approach was proposed in [39] for the development of sustainability-oriented CPPS through the identification of parameters, measures, and data that have pivotal influences on sustainability. As a result, CPPS can be configured in such a way that the environmental impacts are minimized. The authors of [40] proposed a framework architecture that implements a Cyber-Physical Production System for quality prediction and operation control in the metal casting industry. In [41], the authors proposed a Digital Twin (DT)-based decision-making framework for re-scheduling Cyber-Physical Production Systems’ processes.

Other research work efforts have utilized RS for generating personalized PSS recommendations [42,43,44,45]. In [42,43], the authors proposed a multi-criteria recommendation method based on a rough Collaborative Filtering (CF) approach for recommending customized PSS solutions to customers. However, the authors considered only product-service features such as service response time, service cost, service reliability, etc., for providing these PSS customized solutions. They do not consider the structural and quality characteristics of the product itself as a PSS component. In [44], a recommendation framework was proposed to support the various processes of the PSS customization lifecycle described in [4]. A set of recommendation capabilities are identified for each process while considering the various stakeholders’ perspectives. In [45], the authors proposed a hybrid knowledge-based recommender for recommending previously customized PSS variants from a wide range of available ones. The proposed approach integrates two techniques: (i) constraint modeling, where the problem of selecting previously customized PSS variants is modeled as a Constraint Satisfaction Problem (CSP) to filter out PSS variants that do not satisfy constraints (i.e., customer requirements), and (ii) a weighted utility function is adopted to rank the remaining PSS variants based on their utility to the customer. The proposed approach considers customers’ requirements (e.g., functional, structural, quality, environmental, and cost) for the product and its related services when generating PSS recommendations.

Table 1 summarizes the related work on manufacturing recommendation approaches.

According to this summary, most of the previous research has focused on recommending manufacturing services (e.g., capabilities, resources, etc.) required to complete a specific manufacturing task in the cloud manufacturing domain. Many research efforts have used advanced data analytics techniques to predict product quality or to visualize production progress. Furthermore, there have been few research efforts devoted to recommending customized PSS solutions; however, they do not consider assisting customers in selecting the appropriate types of sensors and their adequate locations in service customization as a sub-process of the PSS customization process.

### 2.2. Product Usage Information Exploitation Domains

Product Usage Information (PUI) has been utilized for a variety of purposes in a variety of domains. Influential related work efforts have utilized product usage information as enablers of PSS design improvements [7,46,47,48,49].

In [7], the authors proposed a process and a framework for integrating PUI for PSS development. The applicability of the proposed framework is demonstrated by its application to a car-sharing case study. Similarly, in [46], the authors proposed an approach for using PUI collected from sensors and customer feedback to improve the design of future product generations. The linkage between product structure and PUI is achieved by mapping design parameters to parameters and values provided by sensors.

In [47], the authors proposed a method for connecting product usage information with future product and service development. The proposed method extends the quality table in the Quality Function Deployment (QFD) methodology by incorporating “usage’s quality required by manufacturer” and “quality element of user” as usage information.

The authors in [48] have presented an analysis of the impact of IoT technologies on the PSS provision. By using multiple use cases, the authors discovered that IoT technologies have a significant impact on the various phases of the PSS lifecycle. In [49], a procedure for product-service systems called “Informatization” was proposed. This procedure allows for the analysis of customer data generated during the product usage phase to identify new customer needs and offer new services.

Some related work efforts have utilized collected data along the product’s life cycle, to design and produce products with minimal environmental impact, a method known as Life Cycle Engineering (LCE) [50]. In [51], the authors utilized the LCE approach for lightweight component development in the automotive domain. The authors of [52] proposed an LCE approach for material selection to improve material performance in a specific application while ensuring that it had a minimal environmental impact. In [53], the authors proposed a reference architecture that introduces the Life Cycle Technology (LCT) concept for maximizing the functionality and lifetime of traction batteries.

Other research efforts have utilized product usage information to improve product performance and evaluate customer requirements’ fulfillment [54,55,56]. In [54], the authors utilized product usage from a secure health communication services provider called “Brightsquid” to gain a better understanding of customers’ needs and determine how well the existing product meets their needs.

In [55], the authors proposed an approach that analyzes operating product data collected by embedded sensors to evaluate customer requirements’ fulfillment. The proposed approach is divided into two phases: (i) product operating data are collected, and customers are classified into segments based on their usage patterns, and (ii) the operating data are analyzed to evaluate the performance of product modules.

In [56], the authors proposed an approach to improve product performance and customer value through utilizing product usage data. In this approach, the customer value is identified and then mapped to the physical structure and the function of the product. Relevant parameters influencing the performance of the product’s function are identified and measured to infer optimization measures. These measures are then realized in the form of changes to the product’s parts.

Table 2 summarizes the purposes of exploiting product usage information in a variety of domains. Based on this summary, it was found that product usage information has been exploited extensively for improving the design of PSSs. Moreover, PUI has been exploited to increase customer value by increasing product performance and evaluating the fulfillment of customer requirements.

To the best of our knowledge, no previous work has considered utilizing DW-based approaches to analyze PUI (e.g., product usage incidents). Moreover, the analysis of PUI has not been utilized to determine the optimum types of sensors and their adequate locations during the service customization process. Our proposed approach, in this paper, utilizes data warehousing concepts for analyzing PUI from similar products to the customer’s product to assist customers in determining appropriate types of sensors and their adequate locations.

### 2.3. Utilizing Data Warehousing for Generating Recommendations

Data warehousing (DW) is a repository that collects data from multiple heterogeneous data sources into a single multi-dimensional source for analysis purposes [57]. The main objective of the DW is to improve decision making by providing greater insights into the organization’s performance. The key features of a data warehouse are subject-oriented, integrated, time-variant, and non-volatile. There are three different types of DW architectures that have to be considered when designing a corporate DW which are: single-tier, two-tier, and three-tier. The most common architecture is the three-tier architecture, which is adopted in this paper to design our DW. The following are the main processes carried out for designing the DW using this architecture. First, the data are extracted from multiple external data sources, cleansed, transformed, and loaded in the DW structure using the Extract-Transform-Load (ETL) process. The data are loaded in the DW using a unified multidimensional data model (e.g., star schema, snowflake schema, or fact constellation schema). In this paper, a snowflake schema model is used for structuring the DW due to the nature of the available data. Following that, data analytics and Online Analytical Processing (OLAP) are used to analyze the data in the DW. OLAP is a process that utilizes the DW for multidimensional analysis through the use of data cube operations, such as rollup, drill-down, slicing, and dicing on fact tables and dimensions [58].

In addition to the aforementioned recommendation techniques (cf. Section 2.1), data warehousing concepts have been utilized for generating recommendations and creating RSs in many applications such as movies [15,59], websites [60], books [61], tourism [62], and Geographical Information Systems (GIS) [63].

In [15], the authors utilized data warehousing concepts for creating a movie recommendation system. To recommend a movie to the user, a multi-criteria candidate selection is used, in which movies with genres matching the user’s preferences are recommended. Similarly, in [59], the authors extended traditional approaches to recommender systems by making recommender systems work in multidimensional settings. They extended the traditional two-dimensional user-item environments with other dimensions, such as time and place dimensions.

In [60], the authors presented a data warehouse-based recommender for website recommendations called AWESOME (Adaptive Website Recommendations). The system utilizes a large number of recommenders for generating website recommendations. The authors utilized data warehousing technology and precomputation of recommendations to support scalability and quick web access times. While in [61], the authors proposed a multidimensional recommendation model that integrates contextual information and uses OLAP and data warehousing capabilities for solving book recommendation problems, such as contradicting tribulations hierarchy ratings.

In [62], the authors proposed a DW-based recommendation system to help tourism managers and pilots in the soaring community make soaring decisions by providing accurate and timely information. Moreover, in [63], the authors proposed a Spatial OLAP (SOLAP) recommendation approach that assists users in exploiting spatial data warehouses and retrieving relevant information by recommending spatial MultiDimensional eXpressions (MDX) queries. The proposed approach detects the user’s preferences by comparing the current user’s preferences to the preferences of previous data warehouse users. Queries launched by the user over the SOLAP system are used for analyzing user’s preferences. A similarity measure is then used to measure the similarity between SOLAP users based on their launched MDX queries. Finally, MDX queries are recommended to the current user based on the similarity results.

Table 3 summarizes the related work efforts of using data warehousing for generating recommendations. Based on this summary, it was discovered that data warehousing capabilities have not been utilized effectively for generating recommendations in the manufacturing domain.

## 3. Case Study

According to the latest trend in PSS, customers are increasingly looking for personalized and customized products and services to meet their specific needs. PSS customization entails configuring products with varying degrees of differentiation to meet the needs of various customers. This is combined with service customization, in which customers, with the assistance of product designers or product engineers, expand configured products in the spirit of adaptive customization to include smart IoT devices (e.g., sensors) to improve product usage and facilitate the transition to smart connected products.

The concept of PSS customization is gaining significant interest. However, there are still numerous challenges that must be addressed when designing and offering customized PSSs, such as choosing the optimum types of sensors to install on products and their adequate locations during the service customization process. Accordingly, this necessitates the use of novel techniques to assist customers, with the help of stakeholders from an Original Equipment Manufacturer (OEM), such as product designers, in selecting the appropriate types of sensors to install on their machines and their adequate locations during the service customization process.

Large volumes of usage incident data (e.g., cracks, leaks, breakdowns, etc.) from products (e.g., milling machines, laser cutting machines, etc.) that have been used by various customers, and similar to the one that the customer wishes to expand by adding smart sensors, can be collected during the use phase of these products, while taking into account that these products have been used by customers with the same business environment and business profile as the target customer. In this paper, we focus on incidents whose causes fall under the category of “abnormal machine operating conditions”. Examples of incident causes that fall under the category of “abnormal machine operating conditions” are “extreme temperature”, “excessive rotation speed”, etc.

By analyzing these data, the critical parts with the highest number of incidents, the causes of their incidents, and the influential parts responsible for the occurrence of these incidents can be identified. Sensor types are then suggested to the target customer based on the causes of incidents that occurred on these critical parts.

**Figure 1:**
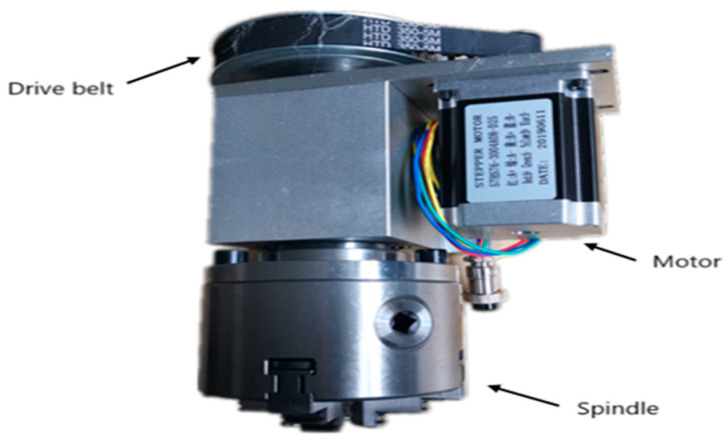
Rotary spindle unit of a CNC milling machine. (Source: https://www.dec-motor.com/, accessed date: 4 December 2021).

In the context of PSS customization, OEM receives multiple orders from various customers who are interested in configurable products (e.g., laser cutting machines, milling machines, etc.) based on their specifications and preferences. Customers may then request that these configured products be expanded by adding smart sensors, which is the scope of our paper. In this section, we present an illustrative case study that considers a Computer Numerical Control (CNC) belt-driven metal milling machine as one of several products that need to be expanded by customers to include smart sensors with the help of product designers. We concentrated our efforts on the rotary spindle unit, which is one of the components of this machine. The considered rotary spindle unit is made up of a motor, a spindle, and a drive belt [64,65] (cf. Figure 1). The motor is positioned next to the spindle. This motor provides rotation and power to the spindle, and the drive belt is used to transmit torque to the spindle shaft. The rotary spindle unit has been built into thousands of similar milling machines.

Assume that the customer (e.g., aerospace engine manufacturer) is interested in expanding this milling machine, in the spirit of adaptive customization, by adding smart sensors (e.g., temperature sensor, pressure sensor, etc.). The customer uses a web application to specify some information about the product that she needs to customize (e.g., product code). She may also specify some information about the nature of her business environment (e.g., business environment temperature, business environment humidity, etc.), and her business profile (e.g., business size, business type). For example, the customer may determine that the temperature nature of her business environment is high, and her business type is metal milling.

By utilizing our proposed recommendation approach, usage incident data generated during the use phase of similar milling machines to the one that the customer wishes to customize by adding smart sensors are collected and analyzed using data warehouse capabilities to identify the most critical parts with the highest number of incidents, the causes of these incidents, and the influential parts responsible for the occurrence of these incidents that occurred on those critical parts. Accordingly, these critical parts are suggested to the target customer as the most important parts to where sensors should be installed in her current milling machine. Sensor types (e.g., temperature sensor, pressure sensor, rotation speed sensor, etc.) are then suggested to the target customer based on the causes of incidents that occurred on these critical parts. Due to the fact that each sensor type suggested to the customer may have several instances, instances of each suggested sensor type are then ranked based on their utility dimensions (e.g., reliability, performance, etc.) and recommended to the customer using a weighted utility function, while taking into account the customer’s interest in each utility dimension in terms of importance weight. The values of these importance weights are directly acquired from the customer during the recommendation session. The customer may indicate that she is interested in reliability with a weight of 0.5.

For example, assume that the “drive belt” is identified as the top-critical part with the highest number of incidents (e.g., cracks), the cause of this incident is “excessive rotation speed”, and the neighboring influential part responsible for the occurrence of these incidents on the “drive belt” is the “*spindle*”, then the suggested sensor type is “*rotation speed sensor*” and it is suggested to be placed on the “*spindle*” as it is the part responsible for the occurrence of these incidents on the drive belt. After that, instances of the suggested rotation speed sensor type are sorted in descending order based on their utility dimensions (e.g., reliability, accuracy, etc.) and the customer’s interest in each utility dimension. Finally, a list of top-k instances is recommended to the customer, implying that the instance with the highest utility value is the best and should be chosen by the customer.

By incorporating our proposed recommendation approach, the customers, with the help of product designers, can decide effectively and accurately which types of sensors to install on their machines and where to place them. This, in turn, lowers the cost of randomly placing sensors on the desired machine.

## 4. Proposed Data Warehousing-Based Recommender Architecture

Service customization in PSSs entails expanding existing customized products by adding smart sensors or IoT communication devices in general to regularly monitor products’ functions. However, selecting the appropriate types of sensors and their adequate locations is a challenge that customers are unable to manage effectively [5]. Consequently, in this section, we propose a DW-based recommender that assists customers in determining the appropriate sensor types to install on their machines and their adequate locations. The proposed RS utilizes large amounts of product usage information, specifically product usage incidents, from similar products to the one the customer wishes to expand by adding smart sensors. By leveraging DW capabilities to analyze these large volumes of product usage incidents (e.g., cracks, leaks, faults, and breakdowns), the parts with the highest number of incidents (critical parts), the causes of their incidents, and the neighboring influential parts responsible for the incidents that occurred on the critical parts can be identified. This provides an answer to (Q1) *how can data analytics techniques be used to support customers in making informed decisions during the customization of services process?* Based on this analysis and the failure modes at hand, sensor types for monitoring these critical parts can be determined and recommended to the customer, providing an answer to (Q2) *how can PUI, particularly product usage incidents, be exploited to assist customers in making informed decisions during the customization of services process?*

**Figure 2:**
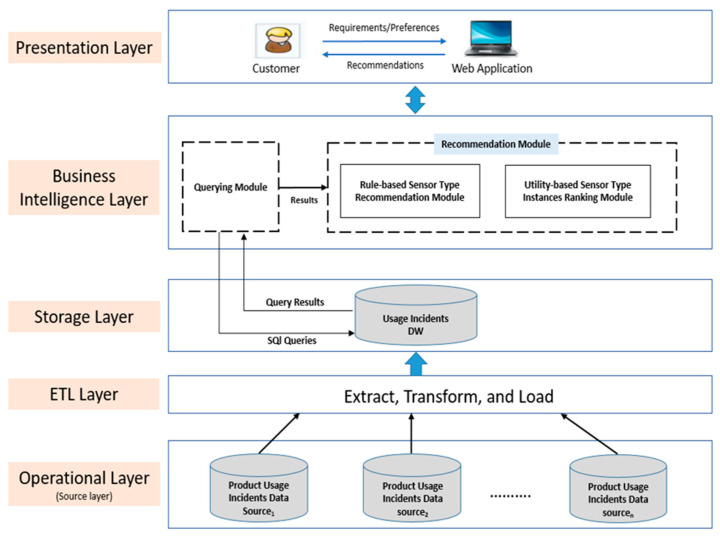
Proposed DW-based recommender architecture (inspired by the typical three-tier data warehouse architecture proposed in [57]).

The architecture of the proposed DW-based recommender is depicted in Figure 2. The proposed RS architecture consists of five layers: the operational layer, the Extract-Transform-Load (ETL) layer, the storage layer, the business intelligence layer, and the presentation layer. These layers are discussed in detail in the following sub-sections.

### 4.1. Operational Layer

This layer is regarded as the source layer, it considers the heterogeneous operative usage information source databases, more specifically usage incidents’ source databases.

### 4.2. ETL Layer

This layer extracts data from the operational layer’s operative usage incidents’ source databases. These data are then filtered, cleaned, and finally loaded into the data warehouse. The data are extracted from the operational layer’s source databases regularly, triggered by incidents (e.g., cracks, breakdowns).

### 4.3. Storage Layer

The purpose of this layer is to load data extracted from the source databases into the data warehouse using a unified multidimensional data model (e.g., star schema, snowflake schema, or fact constellation schema). In this paper, a snowflake schema model is used for structuring the DW due to the normalized nature of its dimensions.

**Figure 3:**
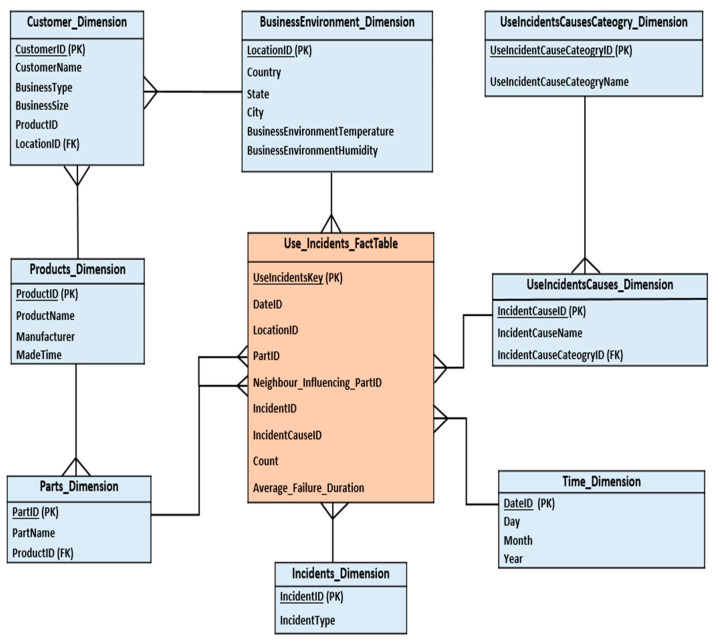
Proposed DW schema.

As shown in Figure 3, the proposed DW snowflake schema consists of eight dimensions which are: *Incidents*, *Parts*, *Products*, *UseIncidentsCauses*, *UseIncidentsCausesCategory*, *BusinessEnvironment*, *Customer*, and *Time*. It also contains one fact table named *Use_Incidents_Facttable*. We briefly describe the components of the DW schema as follows:

- Incidents_Dimension: contains all basic data about product usage incidents. Members of this dimension are IncidentID, and IncidentType (e.g., crack, leak, breakdown, etc.).
- Parts_Dimension: contains all basic data about products’ parts. Members of this dimension are PartID, PartName, and ProductID. According to the context of the case study (cf. Section 3), some examples of part names are drive belt, motor, spindle, etc.
- Products_Dimension: contains all basic data about products. Members of this dimension are ProductID, ProductName, Manufacturer, and MadeTime. Some examples of these products are CNC milling machines, laser cutting machines, etc.
- UseIncidentsCauses_Dimension: contains all basic data about causes of incidents. Members of this dimension are IncidentCauseID, IncidentCauseName (e.g., excessive spindle rotation speed, the extreme temperature of the motor, human mistakes, materials do not meet standards, etc.), and IncidentCauseCateogryID.
- UseIncidentsCausesCateogry_Dimension: stores information about the categories of usage incidents causes. Members of this dimension are UseIncidentCauseCateogryID, and UseIncidentCauseCateogryName (e.g., abnormal machine operating conditions, human behavior, material defect, etc.). In this paper, we focus on incidents whose causes fall under the category of “*abnormal machine operating conditions*”. Some examples of incidents’ causes that fall under the category of “*abnormal machine operating conditions*” and within the context of our case study provided in Section 3 are excessive spindle rotation speed, extreme motor temperature, etc.
- BusinessEnvironment_Dimension: contains information about the customer’s business environment nature in which she uses the product. Members of this dimension are LocationID, Country, State, City, BusinessEnvironmentTemperature (e.g., high temperature), and BusinessEnvironmentHumidity (e.g., high humidity).
- Customer_Dimension: contains information about the customers who use the products and their business profiles. Members of this dimension are CustomerID, CustomerName (e.g., aerospace engine manufacturer X), BusinessType (e.g., milling steel), and BusinessSize (e.g., large).
- Time_Dimension: stores a time hierarchy with levels of Day, Month, and Year.
- Use_Incidents_FactTable: stores information about the products’ parts usage incidents. The “*Use_Incidents*” fact table includes the following measures: Count, and Average_Failure_Duration. For a certain product that is used by a specific customer in a specific business environment, the “count” measure computes the number of incidents that occurred on each product part due to a specific incident cause during a given time interval. The “Average_Failure_Duration” measure computes the average failure duration of a specific incident type that occurred on a specific product part due to a specific incident cause.

### 4.4. Business Intelligence Layer

In this layer, the usage incident data loaded in the DW is utilized to analyze usage incidents that occurred on products similar to the target customer’s product. Based on this analysis, we identify the parts with the highest number of incidents (critical parts), the causes of their incidents, and the neighboring influential parts that are responsible for the occurrence of these incidents on those critical parts. As a result, these critical parts are suggested to the target customer as the most important parts to where sensors should be placed in her current product. Following that, appropriate sensor types are recommended to the target customer based on the causes of incidents that occurred on these critical parts. This layer contains two modules: (i) the querying module, and (ii) the recommendation module. We explain these modules in detail in the rest of this sub-section.

#### 4.4.1. Querying Module

The querying module’s primary function is to retrieve data from the data warehouse based on the query that is submitted to it. The main purpose of our proposed RS is to assist customers in determining the appropriate sensor types to install on their products (e.g., milling machine) and their adequate locations by utilizing usage incidents data (e.g., cracks, leaks, etc.) from similar products to their products.

**Figure 4:**
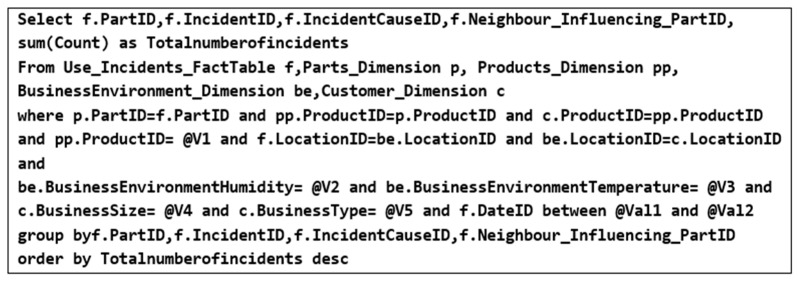
SQL query for retrieving the number of incidents on each product’s part.

Therefore, the usage incidents DW is queried to obtain the number of incidents that occurred in each part of products that are similar to the target customer’s product while taking into account that these products were used by customers with the same business environment and business profile as the target customer. The SQL query in Figure 4 is used to retrieve the required data, taking into consideration that @ V1, @V2, @Val1, and @Val2 are variables that can be changed based on customer requirements.

Based on the query result, the top-k parts with the highest number of incidents, their influential parts, and the causes behind these incidents are then passed to the recommendation module to generate recommendations.

In the context of the case study provided in Section 3, assume that the customer (e.g., aerospace engine manufacturer) indicates that the milling machine that she needs to expand has the code “P111”. She also specifies that her business environment temperature nature is “high” and her business size is “large”. Based on these specifications, usage incidents data from similar milling machines to the target customer’s milling machine and have been used by customers with the same business environment and business profile as the target customer are aggregated and analyzed. After that, these data are queried to identify the number of incidents that occurred on each part, the causes of these incidents, and the neighboring influential parts that are responsible for the occurrence of these incidents using the SQL query mentioned earlier (c.f. Figure 4). Assume that the results of this query are as shown in Table 4.

Based on the query results depicted in Table 4, the IDs of the top two parts with the highest number of incidents, the IDs of incidents that occurred on them, the IDs of the causes of incidents that occurred on them, and the IDs of their neighbor influential parts are then passed to the recommendation module to generate recommendations.

#### 4.4.2. Recommendation Module

The recommendation module is used to suggest appropriate sensor types to the customer based on the results retrieved from the querying module. This module integrates two techniques for generating recommendations: (i) A set of business rules (i.e., if–then rules) is applied to the results retrieved from the querying module, to suggest appropriate sensor types to the customer. (ii) A weighted utility function is applied to rank available instances of each suggested sensor type to the customer based on their utility to the customer. These two techniques are discussed in detail as follows.

1. Rule-Based Sensor Type Recommendation

Using this technique, a set of business rules (e.g., if–then rules) is applied to the query results retrieved from the querying module (cf. Section 4.4.1) to determine the appropriate types of sensors to be suggested to the customer.

According to the context of our case study and the query results depicted in Table 4, the IDs of the top critical parts with the highest number of incidents are selected, which in our case, are 1 and 2. Following that, we use SQL queries to get the name of each critical part’s ID, the incident cause name of its associated incident cause ID, and the neighbor influential name of its associated neighbor influential part ID (cf. Table 5).

To determine the appropriate types of sensors (e.g., rotation speed sensor, temperature sensor, etc.) to be recommended to the customer (e.g., aerospace engine manufacturer), a set of *if–then* rules is then applied to the causes of those incidents that occurred on critical parts. These rules are written in the (**IF** condition **Then** action) format. The condition clause serves as a constraint, whereas the action clause serves as a decision or advice.

Based on the results shown in Table 5, the “*drive belt*” is the top critical part with the most incidents of the type “crack”, and the cause behind these cracks is the “*excessive rotation speed*” of the “*spindle*” part, therefore, a “*rotation speed sensor*” is then suggested to the customer using these rules (i.e., if–then rules). The location of the suggested sensor type is finally determined based on whether or not these incidents were caused by a neighboring influential part. Accordingly, the customer is advised to install the suggested rotation speed sensor on the spindle part as it is the part responsible for the occurrence of these incidents on the drive belt.

In addition, the “*motor*” has been identified as the second top critical part with the highest number of incidents of the type “breakdown”, and the cause of these incidents is “*extreme temperature*”. Therefore, a “*temperature sensor*” is then suggested to the customer (via if–then rules), and the customer is advised to place it on the motor itself, as there is no influential neighbor part responsible for the occurrence of these incidents.

**Figure 5:**
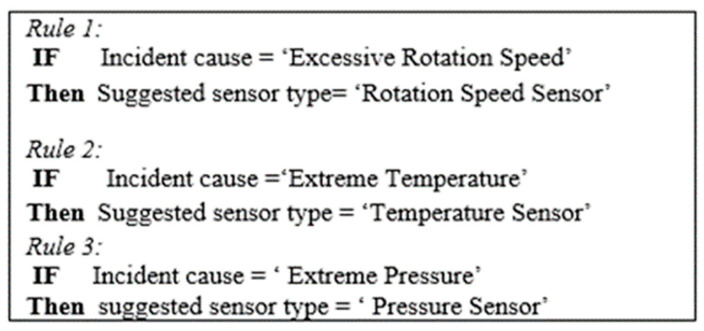
Examples of sensor type determination rules.

Examples of such rules are shown in Figure 5. For example, “*Rule 2*” means that when the cause of a certain incident (e.g., crack, leak, etc.) is “Extreme Temperature”, then the suggested sensor type to the customer is “Temperature Sensor”.

Algorithm 1 illustrates the steps of recommending appropriate sensor types to the customer, as well as their suggested locations. The input to this algorithm is the *usage information* (i.e., usage incidents) from similar products to the target customer’s product while taking into account that these products were used by customers having the same business profile and business environment as the target customer, some information about the target *customer product* (e.g., product code), the target *customer profile*, and her *business environment*.

**Algorithm 1** Appropriate Sensor Type and Location Recommendations**Input:** Usage information (i.e., Usage Incidents) of similar products to target customer’s product, Target customer’s product information, Target customer’s business profile, Target Customer’s business environment.**Output:** Sensor type and location recommendations1: **Begin:**2: Get the Part ID, Incident ID, IncidentCauseID, Neighbor_Influencing_PartID, number of incidents on each part of products similar to the target customer’s product  /* using SQL query in
Figure 4
*/3:    /* using SQL query results at step 2*/4: Critical Parts IDs List (CPL) = Get top-k parts w.r.t number of incidents 5: **for each** Part ID (PID) in (CPL) **do:**6:  Incident Cause ID (IC_ID) = Get PID’s associated IncidentCauseID   /*retrieved at Step2*/7:  Influential PartID (IP_ID) = Get PID’s associated Neighbor _Influencing _Part ID  /*retrieved at step2*/8:  Part Name = get the part name from Parts_ Dimension where PartID = PID   /*using simple SQL query*/9:  Incident Cause Name = Get the incident cause name from UseIncidentsCauses_Dimension where the IncidentCauseID = IC_ID    /*using simple SQL query*/10: Use sensor type determination rules to suggest the sensor type based on the incident cause name /*rules in
Figure 5*/11: **If** (IP_ID is not equal Null) **then**12:  Neighbor_Influential_Part Name = Get the neighbor influencing part name from Parts_Dimension where PartID = IP_ID    /*using simple SQL query*/13:  Suggested sensor location = Neighbor_Influential_Part Name  /*sensor is suggested to be placed on the neighbor influential part*/14: **else**15: Suggested sensor location = Part Name  /* sensor is suggested to be placed on the critical part itself*/16: **end for**17: **return** recommended sensor type and its suggested location18: **End**

First, for products similar to the target customer’s product, the algorithm retrieves the number of incidents that occurred on each part, the IDs of incidents that occurred on them, the IDs of the causes of incidents that occurred on them, and the IDs of their neighboring influential parts that are responsible for the occurrence of these incidents on each part (Step 2). Based on the results obtained in step 2, the IDs of the top-k critical parts with the highest number of incidents are chosen (Step 3).

After that, the algorithm retrieves the name of each critical part’s ID, the incident cause name of its associated incident cause ID retrieved in step 2, and the neighbor influential part name of its associated influential part ID retrieved in step 2 (Step 5 to Step 9). Then, a set of rules (if–then rules) is applied to the incident cause name to determine the suggested sensor type (Step 10). The suggested sensor type location is finally determined based on whether or not these incidents were caused by a neighboring influential part (Step 11 to Step 15).

2. Utility-Based Sensor Type Instances Ranking

Based on the fact that each sensor type suggested to the customer may have several instances, thus we utilize a weighted utility function to rank these available instances based on their utility to the customer. This function is based on the Multi-Attribute Utility Theory (MAUT) approach [66]. To calculate the utility of available sensor type instances, it is important to have prior knowledge about: (i) Contributions of sensor instances in a set of utility dimensions/attributes. Sensor quality attributes (e.g., reliability, accuracy, performance, etc.) that are captured using the product-service blueprint as part of the manufacturing blueprints, which is discussed in detail in Section 5, are utilized for this purpose, and (ii) the weight/importance of each utility dimension based on the customer’s interest.

Based on this information, we applied the following weighted utility function [66] (cf. Equation (1)) to rank available sensor type instances.

(1) $$utility\left(S\right)=\overset{n}{\underset{i=1}{\sum }}w_{i}s_{i}\left(S\right)$$

where *n* is the number of utility dimensions, utility(S) represents the utility of a sensor type instance,$\;w_{i}$ represents the customer’s interest in terms of weight in a utility dimension i, and $s_{i}\left(S\right)\;$is the contribution of sensor type instance (*S*) to a utility dimension i. The values of $w_{i}\;$are acquired from customers directly during the recommendation session.

Algorithm 2 shows the steps of ranking available instances of each suggested sensor type based on their utility to the customer. The input to this algorithm is a list of sensor types that was suggested to the customer, and the customer’s interest in the sensor’s utility dimensions. First, we obtain all available instances of each sensor type recommended to the customer (Step 3). The utility of each sensor type instance is then calculated using Equation (1) (Step 5). Eventually, instances of each suggested sensor type are sorted in descending order based on the utility, and a list of top-k instances is recommended to the customer to choose from.

**Algorithm 2** Utility-Based Sensor Type Instances Ranking**Input:** Suggested sensor types list STL = {*ST_1_, ST_2_, ST_3_, …… ST_n_*}, Customer interest in each utility dimension (Weights)**Output:** Top-K sensor type instances1: **Begin:**2: **for each** suggested sensor type (*ST*) in STL **do:**3:  Sensor Type Instances (STI) = Get sensor type instances4:  **for each** instance (*I*) in STI **do:**5:    UtilityList (UL) = Calculate utility of (*I*) using Equation (1)6:   **end for**7:  Sort instances in (UL) w.r.t. utility descendingly8:  **return** Top-K sensor type instances 9: **end for**10: **End**

### 4.5. Presentation Layer

This layer is considered as the gateway through which the customer interacts with the recommender system to specify her requirements/preferences and receive recommendations.

## 5. Manufacturing Blueprints in Support of Service Customization Recommendations

In Section 4, we provided a DW-based recommendation approach that utilizes large volumes of product usage incidents (e.g., cracks, leaks, etc.) from similar products to the one the customer wishes to expand by adding smart sensors. By utilizing DW capabilities to analyze these large volumes of data, the critical parts with the highest number of incidents, the causes of those incidents, and the neighboring influential parts that are responsible for the occurrence of those incidents on the critical parts are identified. As a result, these critical parts are suggested to the target customer as the most important parts to where sensors should be installed. Following that, appropriate sensor types are determined based on the causes of incidents that occurred on those critical parts. Finally, instances of the suggested sensor types are ranked based on their quality attributes and then recommended to the customer.

However, it is crucial to have knowledge about all available sensor types to be suggested to the customer as well as their quality attributes (e.g., reliability, accuracy, etc.) before generating recommendations. Manufacturing blueprints proposed in [16,17] play a significant role in providing such knowledge and serve as an important component of our recommender system knowledge base. These manufacturing blueprints act as knowledge structures that store extensive product-service and production-related knowledge. Manufacturing blueprints rely on model-based design techniques to manage and interconnect product data, information (both contextual and content), product portfolios and product families, manufacturing assets (personnel, plant machinery, and production line equipment), production processing requirements, and workflows.

Manufacturing blueprints help meet various requirements (e.g., functional, structural, performance, quality, cost, interoperability, time, etc.) of an entire manufacturing network. This information can be collected and placed within a broader operational context, providing the foundation for production actionable “intelligence” and a shift toward more fact-based manufacturing decisions.

Manufacturing knowledge is captured in the following five interconnected knowledge-based structures:

1. *Supplier blueprint* contains knowledge about the capabilities of the supplier’s firm.
2. *Product blueprint* includes detailed information about the product, product parts, and product families. It also includes information about the quality attributes of products and their parts.
3. *Product-service blueprint* provides details about the characteristics of all services that are combined with the physical product. Service types, service-related sensors, and service quality attributes are examples of such characteristics.
4. *Production process blueprint* defines the production processes, the involved activities, and the resources required to accomplish these processes.
5. *Quality assurance blueprint* describes the Key Performance Indicators (KPIs) needed to monitor production processes and troubleshoot production issues.

To provide the customer with a ranked list of the top-k instances of each suggested sensor type based on their utility to the customer, the product-service blueprint proposed in [17] is extended to incorporate appropriate classes that are required to meet customers’ requirements and ease the recommendation process. Some examples of the classes involved in this blueprint are (i) *service*; which describes the service accompanied with the physical product (e.g., maintenance, monitoring); (ii) *service provider*; which refers to the person or company who provides the service; (iii) *sensor*; is a new class added to the product-service blueprint which describes the sensors that can be installed on the physical product; and (iv) *sensor quality attributes*; represents a new other class added to the product-service blueprint that describes the sensor’s quality attributes (e.g., reliability, accuracy, performance, etc.). With the addition of these classes (i.e., sensor, and sensor quality attributes), we are now able to rank available instances of each suggested sensor type based on their utility dimensions and eventually provide them to the customer.

## 6. Implementation and Evaluation

### 6.1. Implementation

**Figure 6:**
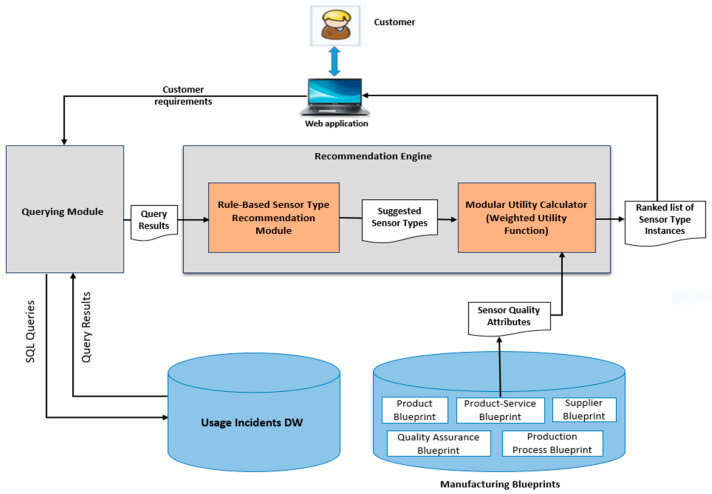
A high-level overview of the RS interacting components.

To ensure the applicability of the proposed DW-based recommender system, a web-based prototype system has been developed as a proof-of-concept based on the proposed system architecture proposed in Section 4. Figure 6 presents a high-level overview of the main components of the proposed RS and the relationships between them. All modules have been developed using open-source tools. The main components of the proposed RS are a set of integrated manufacturing blueprints, usage incidents DW, querying module, and recommendation engine. The proposed DW model for products’ usage incidents was built using SQL Server Management Studio (SSMS) version 2012. We reused implementations of the manufacturing blueprints knowledge base from our previous work [4] and extended them with the manufacturing blueprints extensions provided in Section 5.

**Figure 7:**
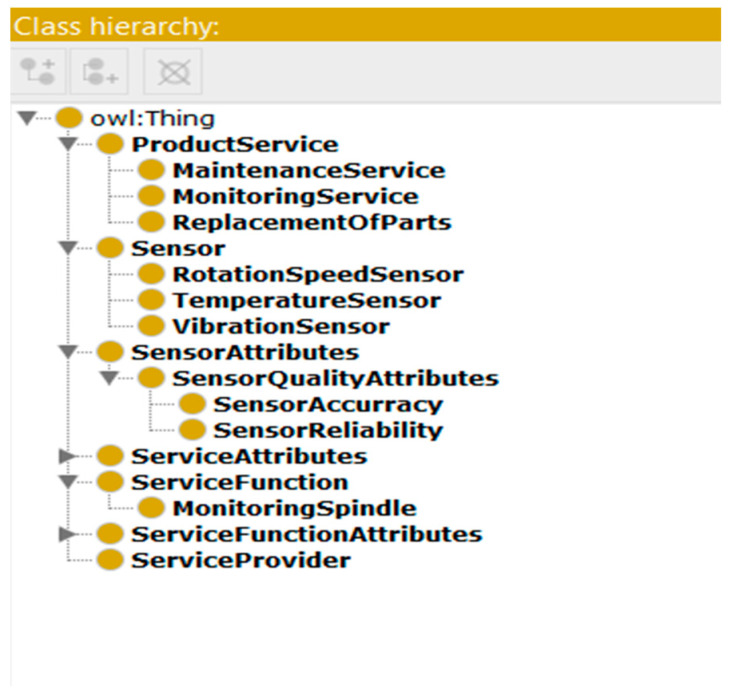
Examples of the classes and sub-classes included in the product-service ontology.

The Ontology Web Language (OWL) standard and Protégé tool-suite [67] were used to implement the extended manufacturing blueprints. The guidelines and steps of how to create ontologies stated by [68] were followed to create the extended blueprints/ontologies discussed in Section 5, which are as follows:

- Creating classes and sub-classes: The implementation of any ontology begins with the creation of classes, which act as the ontology’s main building blocks. To create the product-service blueprint/ontology and its extensions discussed in Section 5, the main classes identified in the top-level ontology such as Service class, Sensor class, Service provider class, service function, etc. are first implemented. After that, the sub-classes related to the domain-level ontology are created. In the context of the case study presented in Section 3, customers are interested in sensors for monitoring their machine’s parts, therefore, the sub-classes of the ‘*Sensor*’ class are ‘*Temperature Sensor*’, ‘*Vibration Sensor’*, ‘*Rotation Sensor*’. Examples of the classes and sub-classes included in the product-service ontology are shown in Figure 7.
- Creating object properties: after creating classes, the relationships among them are defined using object properties. For example, to define that the product service has a sensor, the object property ‘hasSensor’ is used to define the relationship between the ‘ProductService’ class and the ‘Sensor’ class.
- Creating data properties: Data properties are used to create the relationships between an individual and data values. They are used to define the properties of the classes and their data types such as string, int, double, etc. For example, the ‘SensorID’ is defined as one of the data properties of the ‘Sensor’ class and its type is ‘int’.
- Creating instances: The last step for creating ontologies is to create instances of classes included in the hierarchy. The creation of an instance of a class requires: (i) selecting a class, (ii) creating an instance of this class, and (iii) filling the values of the instance slots. For example, an individual instance ‘*TemperatureSensor1′* is created to represent a specific type of the ‘Temperature sensor’ class. After that, its object and data properties are filled.

**Figure 8:**
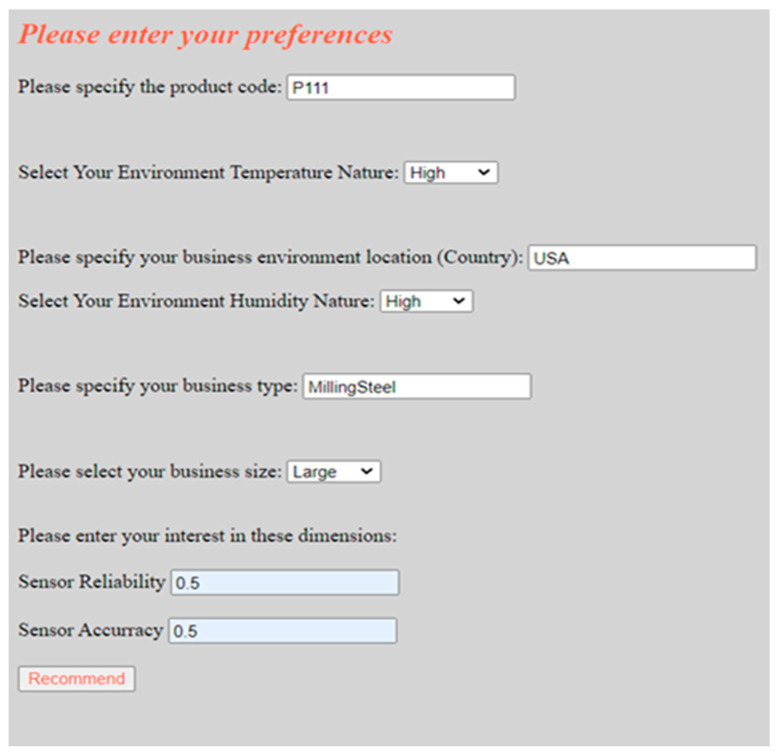
The user interface for the recommender system’s knowledge acquisition.

In order to generate recommendations (e.g., sensors) for the customer (e.g., aerospace engine manufacturer), the following steps are taken during the recommendation process: first, the customer interacts with the RS via a web application to specify some information about the product she needs to customize by adding smart sensors. A screenshot of our recommender system knowledge acquisition user interface is provided in Figure 8. By using this knowledge acquisition interface, the customer can specify some information about the product she wishes to customize such as the product code. The customer may also specify her business type and the temperature nature of her environment. In the context of our case study, the customer indicates that the code of the milling machine she wishes to expand by adding smart sensors is “P111”, her business type is “milling steel”, the temperature nature of her business environment is “high”, and the size of her business is large (cf. Figure 8).

Based on the specifications of the target customer, the DW is then queried to retrieve the critical parts with the greatest number of incidents, the causes of those incidents, and their neighbor influential parts in products similar to the customer’s product. The query results are then passed to the rule-based sensor type recommendation module, a sub-component of the recommendation engine, which employs a set of (if–then) rules to suggest appropriate types of sensors based on the causes of the incidents that occurred on these critical parts. For each suggested sensor type, a modular utility calculator component is used to rank available instances of each suggested sensor type based on their utility to the customer. The inputs to this modular utility calculator component are (i) the customer interest in a set of sensor utility dimensions (e.g., reliability, accuracy, etc.) in terms of weights, which are acquired from the customer during the recommendation session (cf. lower side of Figure 8), and (ii) contributions of the suggested sensor type instances in a set of utility dimensions/attributes.

Finally, the customer is presented with a list of sensor type instances ranked in descending order based on their utility to the customer, implying that the sensor type instance with the highest utility value is the best and should be chosen by the customer.

**Figure 9:**
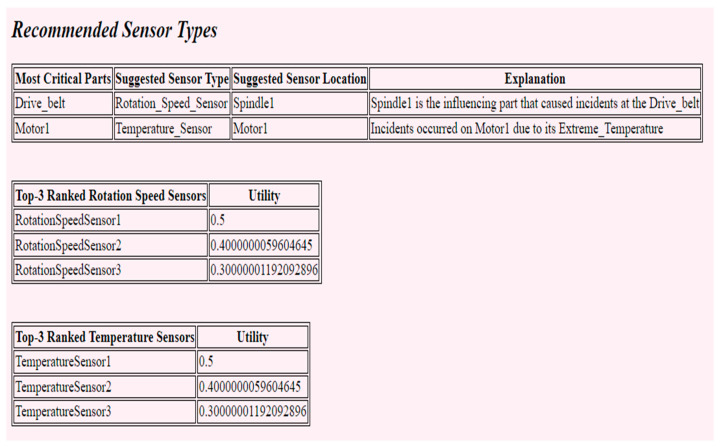
Ranked list of sensor-type instances.

The output of this recommendation process is provided to the customer in a tabular form as shown in Figure 9. According to the query results presented in Table 5 (cf. Rule-Based Sensor Type Recommendation), the identified top critical parts, which are the ‘drive belt’ and the ‘motor’, the suggested sensor types based on the cause of incidents that occurred on each critical part, which are the ‘rotation speed sensor’ and the ‘temperature sensor’, and the suggested location of each sensor, are presented to the customer based on the customer specifications/preferences that are captured using Figure 8 and the data that exists in the DW (cf. upper side of Figure 9). For each suggested sensor type, the customer is then presented with a ranked list of top-3 ranked sensor type instances (cf. center and lower side of Figure 9).

The prototype of the recommender system is implemented as a maven web application using the Eclipse version (4.10.0). Version 8.0 of the Tomcat server was used. Both the front-end and back-end synchronization are handled using Java, JSP, and HTML.

### 6.2. Evaluation

In this sub-section, the performance of the proposed recommender system is evaluated in terms of response time.

#### 6.2.1. Experimental Setting

We set up an experiment with three knowledge bases. These knowledge bases are deployed with different complexity in terms of the size of the DW (i.e., number of rows in dimensions and fact table), and the number of available instances of each sensor type as shown in Table 6. The data used in our DW multidimensional model is synthetically generated using the Datanamic data generator [69]. The knowledge bases are classified into *small*, *medium*, and *large* based on the previously mentioned attributes (i.e., DW size, number of available instances of each sensor type), with the assumption that two critical parts are identified and two sensor types are accordingly suggested to the customer. Our experiment is conducted on Intel ^®^ core ™ i7, with a CPU of 2.7 GHz and 8.0 GB of main memory, under windows 10 pro.

#### 6.2.2. Performance Evaluation Results

The goal of the performance evaluation is to determine how much time the RS takes to calculate and provide recommendations to the customer based on her specifications. Table 7 shows how much time it takes to generate recommendations based on each knowledge base. Based on the performance evaluation results depicted in Table 7, we show that our recommender can provide recommendations even for the large knowledge base within the recommended system response time boundaries presented in [70], which are as follows:

- 100 ms, is the upper limit for keeping users feel that the system reacts instantaneously.
- 1000 ms, is the upper limit for keeping users’ thoughts uninterrupted.
- 10,000 ms, is the upper limit for keeping the focus of the users on the dialogue.

### 6.3. Discussion

In this paper, we proposed a DW-based recommender that assists customers in selecting the appropriate types of sensors to install on their machines and their adequate locations to regularly monitor machines’ functions. The proposed recommendation approach utilized DW capabilities to analyze large volumes of product usage incidents (e.g., cracks, leaks, faults, and breakdowns) from similar products to the one that the customer wishes to expand by adding smart sensors. The analysis of these data helps in identifying the parts with the highest number of incidents (critical parts), the causes of their incidents, and the neighboring influential parts responsible for the occurrence of these incidents on those critical parts. As a result, these critical parts are suggested to the target customer as the most important parts to where sensors should be installed in her current product. Sensor types are then suggested to the target customer based on the causes of incidents that occurred on those critical parts.

A case study that considers the rotary spindle units of a CNC milling machine is used to demonstrate the applicability and utility of the proposed recommendation approach and its implemented solutions.

The main difficulty in validating the proposed approach is the scarcity of large amounts of real-world product usage data sets (e.g., product usage incidents); however, a sample data set is generated synthetically using the Datanamic data generator [69] and used for evaluating the performance of the proposed recommender system in terms of response time. As future work, we plan to test the performance of the proposed system using a large-scale data set, either generated or real.

## 7. Conclusions

Manufacturers are shifting from a traditional product-centric model to a service-centric one by offering not only products, but products accompanied by services. This paradigm is known as Product-Service Systems (PSSs). PSSs’ customization entails two intertwined processes: product customization and service customization. Product customization is the process of configuring products with varying degrees of differentiation to meet the needs of different customers. Service customization, on the other hand, refers to the expansion of customized products via the addition of smart sensors or Internet-of-Things (IoT) communication devices in general.

Condition monitoring sensors are embedded in products or near production systems to monitor physical parameters in machinery such as vibration, temperature, pressure, etc., to detect changes that may indicate a developing fault. However, selecting the appropriate types of sensors and their adequate locations is a challenge that the customers are unable to manage effectively. Moreover, placing sensors randomly by customers may result in cost increases. Accordingly, this creates a demand for the adoption of novel techniques/approaches to assist customers in choosing the optimum types of sensors and their adequate locations.

Therefore, this study aims to address two research questions: (Q1) how can data analytics techniques be used to support customers in making informed decisions during the customization of services process? Followed by (Q2) how can PUI, particularly product usage incidents, be exploited to assist customers in making informed decisions during the customization of services process?

We proposed a data warehouse-based recommender system that helps customers in determining the appropriate types of sensors to install on their machines and their adequate places. The proposed RS utilizes large amounts of product usage information, specifically product usage incidents, from similar products to the one the customer wishes to expand by adding smart sensors. By leveraging DW capabilities to analyze these large volumes of product usage incidents (e.g., cracks, leaks, faults, and breakdowns), the parts with the highest number of incidents (critical parts), the causes of their incidents, and the neighboring influential parts responsible for the incidents that occurred on those critical parts can be identified, providing an answer to the first research question (Q1). Consequently, sensor types for monitoring these critical parts are determined and recommended to the customer based on this analysis and the failure modes at hand, and this provides an answer to the second research question (Q2).

A case study that considers the rotary spindle units of a CNC milling machine is used to demonstrate the applicability and utility of the proposed recommendation approach and its implemented solutions. Moreover, the performance of the proposed recommender system is experimentally evaluated in terms of response time using synthetically generated data. The performance evaluation results depict that our recommender can provide recommendations within the recommended system response time boundaries provided in [70].

Future work efforts are going in parallel and complementary directions: (i) enhancing the DW multidimensional model by incorporating new measures/facts that may improve the recommendation process; (ii) utilizing 3D visualization and domain-specific languages to present sensor recommendations in a user-friendly manner; (iii) enhancing data analytics functions by incorporating advanced data analysis methods (e.g., deep neural networks). Another potential research direction will be dedicated to the realization of the identified recommendation capabilities in [44] for the other processes of the PSS customization lifecycle.

## Figures and Tables

**Table 1:** Related work recommendation approaches in manufacturing.

Paper	Used Techniques	Application Domain	Recommendation Capabilities/ System Capabilities	Evaluation Mechanism
[28]	Clustering algorithm, Support vector machine classification algorithm	Additive Manufacturing	Additive manufacturing design features	R/C car racing components case study
[29]	Social network, Collaborative filtering	Manufacturing	Manufacturing services	Experimental evaluation
[30]	Link structure analysis, User-based CF	Manufacturing	Manufacturing services	Experimental evaluation
[31]	Clustering algorithm, CF approach	Cloud manufacturing	Manufacturing services	Experimental evaluation
[32]	Time-aware targeted reconstructing service descriptions	Cloud manufacturing	Manufacturing services	Experimental evaluation on real-world data set
[33]	Deep neural network approach	Cloud manufacturing	Manufacturing services	Simulated case study
[34]	Three-layer feed-forward neural network	Cloud manufacturing	Manufacturing services	Simulated case study
[35]	Deep Belief Neural Network, regression model	Manufacturing	Suitable design parameters for manufacturing	Experimental evaluation
[36]	Temporal Convolutional Network	Manufacturing	Predictive services (e.g., predictive maintenance strategies)	Packaging machine case study
[37]	Minimal-Redundancy-Maximal-Relevance algorithm, Convolutional Neural Network	Manufacturing	Adapting materials concentration (e.g., penicillin concentration)	Penicillin Fermentation process
[38]	Event stream processing, complex event processing	Cyber-Physical Production Systems (CPPS)	Production monitoring services (e.g., production progress visualization)	Traditional factory case study
[39]	Causal chain analysis	CPPS	Developing sustainable CPPS	3D-printing case study
[40]	Decision tree, random forest, support vector machine	CPPS	Quality prediction and operation control	Metal casting process of an actual piston factory
[41]	Fuzzy inference systems	CPPS	CPPS re-scheduling and optimization	Pilot assembly line
[42,43]	Decision making and trial evaluation laboratory, CF approach	PSS customization	Customized PSS solutions	Elevator case study
[44]	Knowledge-based techniques	PSS customization	Customized PSS solutions, suppliers, production plans	Laser machines case study
[45]	Constraint modeling, weighted utility function	PSS customization	Customized PSS solutions	Laser machines case study

**Table 2:** Related work efforts for exploiting product usage information (PUI).

Paper	Application Domain	Considered PUI	Purpose	PUI Analysis Technique	Evaluation Mechanism
[7]	PSS	Sensor data (e.g., the temperature of brake hoses data)	Improving PSS design	Not provided	Car-sharing case study
[46]	PSS	Sensor data (e.g., temperature data), Customer feedback	Improving PSS design	Statistical measures (e.g., median, mean, etc.)	Washing machine case study
[47]	PSS	Sensor data (e.g., noise, temperature)	Improving PSS design	Quality function deployment (QFD) methodology	Hairdryer case study
[48]	PSS	Data generated from IoT technologies	Improving PSS provision	Not provided	Home delivery solutions case studies
[49]	PSS	Customer data (e.g., customer habits)	Deducing new customer needs, offering new services	Not provided	Not provided
[53]	Manufacturing	Sensor data (e.g., operating temperature)	Improve product life cycle performance	Data mining techniques	All-solid-state traction batteries
[54]	Software requirements engineering	Brightsquid’s various repositories (e.g., JIRA)	Evaluating customer requirements fulfillment	Spearman’s rank-order correlation analysis	Case study on health communication services provider (Brightsquid company)
[55]	Software requirement engineering	Sensor data (e.g., CPU utilization data, battery remaining data)	Evaluating product performance	Statistical measures	Case study on smartphones
[56]	Machining and plant engineering sector	Sensor data (e.g., air volume flow rate, rotation speed)	Improving product performance	Not provided	Compressed air plant case study

**Table 3:** Related work efforts of using data warehousing for generating recommendations.

Paper	Application Domain	Recommendation Capabilities	Evaluation Mechanism
[15]	E-commerce	Movies	Not provided
[59]	E-commerce	Movies	Not provided
[60]	E-commerce	Websites	Experimental evaluation
[61]	E-commerce	Books	Not provided
[62]	Tourism	Appropriate Soaring sites	Not provided
[63]	Geographicalinformation systems	Spatial MDX queries	Experimental evaluation

**Table 4:** Examples of the SQL query results.

Part ID	Incident ID	Incident Cause ID	Neighbor Influencing Part ID	Total Number of Incidents
1	1	1	3	36
2	4	3	Null	24
1	1	2	Null	12

**Table 5:** The critical parts’ names, the types of incidents that occurred on them, and the names of their neighbor influential parts.

Part ID	Part Name	Incident Type	Incident Cause Name	Neighbor Influencing Part Name
1	Drive belt	Crack	Excessive rotation speed	Spindle
2	Motor	Breakdown	Extreme Temperature	Null

**Table 6:** Description of the three knowledge bases used for performance evaluation.

Knowledge Base	DW Size								# of Available Instances of Each Sensor Type	
	Fact Table Size (# of Rows	Products Dimension Size	Time Dimension Size	Parts Dimension Size	Incidents Dimension Size	Incidents Causes Dimension Size	Customer Dimension Size	Business Environment Dimension Size	Rotation Speed Sensor	Temperature Sensor
**Small**	500	200	250	100	100	100	100	100	5	5
**Medium**	750	500	500	200	200	200	200	200	20	20
**Large**	1000	1000	1000	300	300	300	300	300	40	40

**Table 7:** Performance evaluation results.

Knowledge Base	Response Time
Small	3008 ms
Medium	5020 ms
Large	5061 ms

## References

[1] Kuo T.C., Huang S.H., Zhang H.C. (2001). Design for manufacture and design for “X”: Concepts, applications, and perspectives. Comput. Ind. Eng..

[2] Elgammal A., Papazoglou M., Krämer B., Constantinescu C. (2017). Design for Customization: A New Paradigm for Product-Service System Development. Procedia CIRP.

[3] Bustinza O.F., Bigdeli A.Z., Baines T., Elliot C. (2015). Servitization and Competitive Advantage: The Importance of Organizational Structure and Value Chain Position. Res. Manag..

[4] Papazoglou M., Elgammal A., Krämer B. (2018). Collaborative on-demand Product-Service Systems Customization lifecycle. CIRP J. Manuf. Sci. Technol..

[5] Omar Y.M., Minoufekr M., Plapper P. (2019). Business analytics in manufacturing: Current trends, challenges and pathway to market leadership. Oper. Res. Perspect..

[6] Abramovici M., Lindner A. (2011). Providing product use knowledge for the design of improved product generations. CIRP Ann. Manuf. Technol..

[7] Kammerl D., Novak G., Hollauer C., Mörtl M. Integrating usage data into the planning of Product-Service Systems. Proceedings of the 2016 IEEE International Conference on Industrial Engineering and Engineering Management (IEEM).

[8] Mohr D., Camplone G., Wee D., Moller T., Bertoncello M. Car data: Paving the way to value-creating mobility: Perspectives on a new automotive business model. In *Advanced Industries*; McKinsey & Company: 2016. https://www.mckinsey.com/~/media/McKinsey/Industries/Automotive%20and%20Assembly/Our%20Insights/Creating%20value%20from%20car%20data/Creating%20value%20from%20car%20data.as.

[9] Nagorny K., Lima-monteiro P., Barata J., Colombo A.W. (2017). Big Data Analysis in Smart Manufacturing: A Review. Int. J. Commun. Netw. Syst. Sci..

[10] Lu J., Wu D., Mao M., Wang W., Zhang G. (2015). Recommender System Application Developments: A survey. Decis. Support Syst..

[11] Priyanka P. (2017). A Survey Paper on Various Algorithm’s based Recommender System. IOSR J. Comput. Eng..

[12] Bouraga S., Jureta I., Faulkner S., Herssens C. (2014). Knowledge-based recommendation systems: A survey. Int. J. Intell. Inf. Technol..

[13] Chicaiza J., Valdiviezo-Diaz P. (2021). A comprehensive survey of knowledge graph-based recommender systems: Technologies, development, and contributions. Information.

[14] Beheshti A., Yakhchi S., Mousaeirad S., Ghafari S.M., Goluguri S.R., Edrisi M.A. (2020). Towards cognitive recommender systems. Algorithms.

[15] Jakkhupan W., Kajkamhaeng S. Movie Recommendation Using OLAP and Multidimensional Data Model. Proceedings of the 13th IFIP International Conference on Computer Information Systems and Industrial Management.

[16] Papazoglou M.P., Van Den Heuvel W.J., Mascolo J.E. (2015). A reference architecture and knowledge-based structures for smart manufacturing networks. IEEE Softw..

[17] Papazoglou M., Elgammal A. The manufacturing blueprint environment: Bringing intelligence into manufacturing. Proceedings of the 2017 International Conference on Engineering, Technology and Innovation (ICE/ITMC).

[18] Dhelim S., Ning H., Aung N., Huang R., Ma J. (2020). Personality-Aware Product Recommendation System Based on User Interests Mining and Metapath Discovery. IEEE Trans. Comput. Soc. Syst..

[19] Deepak G., Kasaraneni D. (2019). Ontocommerce: An ontology focused semantic framework for personalised product recommendation for user targeted e-commerce. Int. J. Comput. Aided Eng. Technol..

[20] Singh M., Rishi O.P. (2020). Event driven Recommendation System for E-commerce using Knowledge based Collaborative Filtering Technique. Scalable Comput. Pract. Exp..

[21] Zhang Z., Lin H., Liu K., Wu D., Zhang G., Lu J. (2013). A hybrid fuzzy-based personalized recommender system for telecom products/services. Inf. Sci..

[22] Jannach D., Zanker M., Fuchs M. (2009). Constraint-Based Recommendation in Tourism: A Multiperspective Case Study. Inf. Technol. Tour..

[23] Zanker M., Aschinger M., Jessenitschnig M. (2010). Constraint-based personalized configuring of product and service bundles. Int. J. Mass Cust..

[24] Felfernig A., Friedrich G., Jannach D., Zanker M. (2006). An Integrated Environment for the Development of Knowledge-Based Recommender Applications. Int. J. Electron. Commer..

[25] Felfernig A., Isak K., Szabo K., Zachar P. (2007). The VITA financial services sales support environment. Proceedings of the 22nd Conference on Artificial Intelligence: AAAI-07.

[26] Richter M.M., Aamodt A. (2006). Case-based reasoning foundations. Knowl. Eng. Rev..

[27] Felfernig A., Friedrich G., Jannach D., Zanker M. (2011). Developing Constraint-based Recommenders. Recommender Systems Handbook.

[28] Yao X., Moon S.K., Bi G. (2017). A hybrid machine learning approach for additive manufacturing design feature recommendation. Rapid Prototyp. J..

[29] Zhang W.Y., Zhang S., Chen Y.G., Pan X.W. (2013). Combining social network and collaborative filtering for personalised manufacturing service recommendation. Int. J. Prod. Res..

[30] Zhang S., Yang W.T., Xu S., Zhang W.Y. (2017). A Hybrid Social Network-based Collaborative Filtering Method for Personalized Manufacturing Service Recommendation. Int. J. Comput. Commun. Control.

[31] Liu J., Chen Y. (2019). A personalized clustering-based and reliable trust-aware QoS prediction approach for cloud service recommendation in cloud manufacturing. Knowl. Based Syst..

[32] Hao Y., Fan Y., Zhang J. (2019). Service recommendation based on description reconstruction in cloud manufacturing. Int. J. Comput. Integr. Manuf..

[33] Simeone A., Caggiano A., Deng B., Boun L. (2019). A deep learning based-decision support tool for solution recommendation in cloud manufacturing platforms. Procedia CIRP.

[34] Simeone A., Zeng Y., Caggiano A. (2021). Intelligent decision-making support system for manufacturing solution recommendation in a cloud framework. Int. J. Adv. Manuf. Technol..

[35] Bai Y., Li C., Sun Z., Chen H. Deep neural network for manufacturing quality prediction. Proceedings of the 2017 Prognostics and System Health Management Conference (PHM-Harbin).

[36] Brunelli L., Masiero C., Tosato D., Beghi A., Susto G.A. (2019). Deep Learning-based Production Forecasting in Manufacturing: A Packaging Equipment Case Study. Procedia Manuf..

[37] Dong Y., Zhuang Y., Yan X. (2021). Data-Driven Quality Prediction of Batch Processes Based on Minimal-Redundancy-Maximal-Relevance Integrated Convolutional Neural Network. Math. Probl. Eng..

[38] Fang P., Yang J., Zheng L., Zhong R.Y., Jiang Y. (2020). Data analytics-enable production visibility for Cyber-Physical Production Systems. J. Manuf. Syst..

[39] Rogall C., Mennenga M., Herrmann C., Thiede S. (2022). Systematic Development of Sustainability-Oriented Cyber-Physical Production Systems. Sustainability.

[40] Lee J.H., Do Noh S., Kim H.J., Kang Y.S. (2018). Implementation of Cyber-Physical Production Systems for Quality Prediction and Operation Control in Metal Casting. Sensors.

[41] Villalonga A., Negri E., Biscardo G., Castano F., Haber R.E., Fumagalli L., Macchi M. (2021). A decision-making framework for dynamic scheduling of cyber-physical production systems based on digital twins. Annu. Rev. Control.

[42] Song W., Sakao T. (2018). An environmentally conscious PSS recommendation method based on users’ vague ratings: A rough multi-criteria approach. J. Clean. Prod..

[43] Song W. (2018). Personalized Recommendation of Customizable PSS to Customers. Customization-Oriented Design of Product-Service System.

[44] Esheiba L., Elgammal A., El-Sharkawi M.E. Recommendation framework for on-demand smart product customization. Proceedings of the 21st International Conference on Enterprise Information Systems.

[45] Esheiba L., Elgammal A., Helal I.M.A., El-Sharkawi M.E. (2021). A Hybrid Knowledge-Based Recommender for Product-Service Systems Mass Customization. Information.

[46] Lützenberger J., Klein P., Hribernik K., Thoben K.-D. (2016). Improving Product-Service Systems by Exploiting Information from The Usage Phase. A Case Study. Procedia CIRP.

[47] Hara T. (2018). Integrating usage information into quality function deployment for further PSS development. Procedia CIRP.

[48] Sassanelli C., Seregni M., Hankammer S., Cerri D., Terzi S. The role of internet of things (IoT) technologies for individualisation and service quality of a PSS. Proceedings of the Summer School Francesco Turco.

[49] Opresnik D., Hirsch M., Zanetti C., Taisch M. (2013). Information—The Hidden Value of Servitization. IFIP Adv. Inf. Commun. Technol..

[50] Wanyama W., Ertaş A., Zhang H.-C., Ekwaro-Osire S. (2003). Life-cycle engineering: Issues, tools and research. Int. J. Comput. Integr. Manuf..

[51] Kaluza A., Kleemann S., Fröhlich T., Herrmann C., Vietor T. (2017). Concurrent Design & Life Cycle Engineering in Automotive Lightweight Component Development. Procedia CIRP.

[52] Ribeiro I., Peças P., Silva A., Henriques E. (2008). Life cycle engineering methodology applied to material selection, a fender case study. J. Clean. Prod..

[53] Dilger N., Kaluza A., Kiesewetter A., Cerdas F., Blume S., Zellmer S., Herrmann C. (2021). Definition and Reference Framework for Life Cycle Technologies in Life Cycle Engineering—A Case Study on All Solid State Traction Batteries. Procedia CIRP.

[54] Hemmati A., Al Alam S.M.D., Carlson C. Utilizing Product Usage Data for Requirements Evaluation. Proceedings of the 2018 IEEE 26th International Requirements Engineering Conference (RE).

[55] Chen H., Zhang L., Chu X. Performance Assessment of Product Modules Based on Usage Data Collected Through Embedded Sensors. Proceedings of the 2018 IEEE International Conference on Industrial Engineering and Engineering Management (IEEM).

[56] Riesener M., Dolle C., Becker A., Schuh G. Framework for the Continuous Increase of Product Performance by Analyzing Product Usage Data. Proceedings of the 2019 IEEE International Conference on Industrial Engineering and Engineering Management (IEEM).

[57] Inmon W.H. (2005). Building the Data Warehouse.

[58] Romero O., Abelló A. (2009). A Survey of Multidimensional Modeling Methodologies. Int. J. Data Warehous. Min..

[59] Adomavicius G., Tuzhilin A. Extending Recommender Systems: A Multidimensional Approach. Proceedings of the International Joint Conference on Artificial Intelligence (IJCAI-01), Workshop on Intelligent Techniques for Web Personalization (ITWP2001).

[60] Thor A., Rahm E. AWESOME—A Data Warehouse-based System for Adaptive Website Recommendations. Proceedings of the 30th International Conference on Very Large Data Bases.

[61] Tiwari R.G., Husain M., Gupta B., Agrawal A. Amalgamating Contextual Information into Recommender System. Proceedings of the 2010 3rd International Conference on Emerging Trends in Engineering and Technology.

[62] Araque F., Salguero A., Abad M.M. (2006). Application of data warehouse and Decision Support System in soaring site recommendation. Information and Communication Technologies in Tourism.

[63] Aissi S., Gouider M.S., Sboui T., Ben Said L. (2015). A spatial data warehouse recommendation approach: Conceptual framework and experimental evaluation. Hum.-Cent. Comput. Inf. Sci..

[64] Sevic M., Keller P. (2019). Design of Cnc Milling Machine as a Base of Industry 4.0 Enterprise. MM Sci. J..

[65] Ag S. (2009). *The Right Spindle Solution for Any Task*. https://www.yumpu.com/en/document/view/41469622/motors-siemens.

[66] Winterfeldt D., von Edwards W. (1986). Decision Analysis and Behavioral Research.

[67] BMIR Protégé n.d. https://protege.stanford.edu/.

[68] Noy N.F., McGuinness D.L. (2001). Ontology Development 101: A Guide to Creating Your First Ontology.

[69] Datanamic Data Generator, n.d. https://www.datanamic.com/datagenerator/.

[70] Nielsen J. (1993). Usability Engineering.

